# Investigating Orthorexia Nervosa With the ORTO-R in a Sample of University Students With or Without Subthreshold Autism Spectrum: Focus on Dietary Habits and Gender Differences

**DOI:** 10.3389/fpsyt.2022.900880

**Published:** 2022-07-14

**Authors:** Liliana Dell'Osso, Ivan Mirko Cremone, Ilaria Chiarantini, Alessandro Arone, Danila Casagrande, Gabriele Massimetti, Claudia Carmassi, Barbara Carpita

**Affiliations:** Department of Clinical and Experimental Medicine, University of Pisa, Pisa, Italy

**Keywords:** autistic traits, Orthorexia, students, eating disorders, gender difference

## Abstract

**Background:**

The aim of the present study was to investigate the presence of Orthorexia (ON) symptoms in a sample of University students with or without autistic traits (AT), specifically focusing on evaluating the role of gender and of dietary habits in the association between ON and autism spectrum.

**Methods:**

Subjects were requested to anonymously fill out the questionnaires through an online form.

**Results:**

Two thousand one hundred forty students participated in the study. Subjects with significant AT, measured by means of the Adult Autism Sub-threshold spectrum (AdAS Spectrum) reported significantly higher ON symptoms, as measured by ORTO-R scores, than subjects with low AT. Females and subjects following a vegetarian/vegan diet reported significantly higher ORTO-R scores than males and than subjects following an omnivorous diet, respectively. Significant positive correlations were found between ORTO-R and AdAS Spectrum scores. A decision tree model, with gender, type of diet and presence of high AT as independent variables and ORTO-R score as dependent variable, showed in the first step the presence of significantly higher ORTO-R scores among females than among males, and in the second step showed in both genders the presence of higher ORTO-R scores among subjects with high AT than in those with low AT. A significant interaction of gender and presence/absence of high AT was reported on ORTO-R score, with a higher increasing trend of ORTO-R score with the increase of AdAS Spectrum score among females than among males.

**Conclusions:**

Our results further highlighted the association between AT and ON, in particular among females.

## Introduction

During the last decades, in parallel with the evolution of social and cultural environment, modern psychiatry faced the emergence of new psychopathological conditions, characterized by peculiar clinical pictures. This is the case of Orthorexia nervosa (ON), a condition described for the first time by Steve Bratman in the late 1990s (1). ON features an eating pattern mainly characterized by an abnormal fixation and obsession for a healthy style of eating (1). The word “orthorexia” derives from the Greek “ortho,” which means “correct/adequate,” and “orexis,” which means “appetite.” Individuals affected by this condition usually follow restrictive dietary habits, progressively excluding more and more foods considered unhealthy from their diet. Despite the persistence of diagnostic uncertainty for this condition, which to date is not included in the fifth edition of the Diagnostic and Statistical Manual of Mental disorders (DSM-5) (2), a wide range of possible risk factors has been proposed, such as an individual or family history of other mental disorders, previous overweight or obesity, neurotic or perfectionist personality traits or also the presence of a high income (3–9). Other features associated with ON were the previous presence of specific dietary habits, such as veganism or vegetarianism, a high income or post-graduate education (despite further authors reported instead as a risk factor the presence of lower levels of education—below senior high school level) (3–9). In addition, specific populations, such as athletes or health workers, seem to show a greater risk toward the development of ON symptoms (3–9). According to previous studies, the prevalence of ON may vary depending on the sample considered, ranging from 1 to 57.6% in the general population and from 35 to 57.8% in high-risk groups, such as athletes or students of health/nutritional sciences (3, 5, 6, 10–12). However, it should be noted that some of these studies measured ON prevalence by means of rather unreliable tools, more oriented to assess the presence of thoughts and behaviors related to healthy eating than a proper ON condition. The difference in ON distribution depending on gender is still debated. Several studies reported a higher prevalence among females, although with a lower male/female ratio with respect to AN (3, 5, 6, 13–15). However, not all the studies confirmed this result (16, 17). Several instruments have been developed for measuring ON. The most frequently employed questionnaire in this field is the ORTO-15 (15, 18), although this instrument has been increasingly criticized due to the lack of reliability and adherence to the ON symptoms. In particular, the ORTO-15 was developed and validated several years ago, before a more shared agreement on ON definition and features was reached in the scientific literature (19). Only recently Rogoza and Donnini (19) proposed a revised version of the instrument, the ORTO-R, which was developed with the aim to be in line with the more updated descriptions of ON, reporting promising results in the validation study.

Some authors suggested that the spreading of ON may mirror the spreading of the contemporary ideal of healthy eating, which is gradually replacing the ideal of thinness typical of the 1980s and 1990s: in this framework, ON might be considered a restrictive eating disorder in the same psychopathological spectrum of restrictive Anorexia nervosa (AN-R), with different presentations linked to environmental factors (5, 20, 21). Several studies, in line with this hypothesis, highlighted the similarities between AN and ON, stressing that, despite the shift of the focus on food quality rather than food quantity, these conditions would share a common symptomatology core and would also be associated with similar personality traits, such as perfectionism or inflexibility (14, 20). On the other hand, ON was also hypothesized to be associated with other psychopathological dimensions, such as the obsessive-compulsive spectrum, stressing the presence of rituals, repetitive behaviors and perfectionism traits in both the disorders (4). At the same time, increasing literature reported the presence of a possible association between AN and female phenotypes of Autism Spectrum Disorder (ASD). ASD is a neurodevelopmental disorder characterized by an impairment in social communication and interactions, narrow interests and repetitive behaviors, which shows a higher prevalence among males (male/female gender ratio about 4–1), while AN shows instead a greater prevalence among females (2). Since the '70s, some authors reported a familial aggregation between AN and ASD as well as similar clinical features (14, 20, 22). It was pointed out that the strict focus on food and diet and the ritualized behaviors linked to food intake typical of AN may resemble the pattern of narrow interests and repetitive behaviors of ASD more than the obsessions and compulsion of obsessive-compulsive disorder (14). In addition to a pattern of rigid habits, inflexibility and narrow interests, the presence of deficits in socio-emotional reciprocity and alterations in theory of mind was also reported among AN subjects, further stressing its symptomatological overlaps with the autism spectrum (14, 23–25). Several studies in the last years highlighted, among people with AN, a higher prevalence of ASD (26). The presence of sub-threshold autistic traits (AT) was reported to be particularly higher among subjects with AN (14, 21), although increased AT were also found in subjects with Bulimia nervosa and Binge eating disorder, which were often been associated with mood disorder spectrum in the literature (14, 21, 27).

It should be noted that the label of “AT” is used for describing the presence of symptoms and traits similar, although sub-threshold, to those reported by subjects with ASD. In line with the spectrum model, which features a dimensional approach to psychopathology and recognizes the presence of a continuum between full-fledged clinical disorders and isolated, atypical or more nuanced symptoms (28), AT seem to be distributed in a continuum from the general to the clinical population, being particularly represented in some specific high risk groups, including subjects with other psychiatric disorders (21, 29–37). The similarities between AN and autism spectrum, together with the inverse gender prevalence of these conditions, led some authors to hypothesize that AN might be re-conceptualized as a female-specific phenotype of ASD (15). The possible link between autism spectrum and AN was further supported by studies focused on female presentations of ASD, which reported that autistic-like symptoms may be under-recognized among females due to the presence of different features with respect to those typical of males. In particular, ASD females seem to report a milder impairment in social relationships, showing a higher ability to cope with the social environment through camouflaging strategies, although reporting more often social anxiety symptoms (38–40). Moreover, female forms of ASD would feature different restricted interests, such as spending time with animals, focusing on fictions or celebrities, or also on food and diet (15, 39, 40). Despite the large amount of studies about the link between autism spectrum and AN, limited research focused on evaluating the possible association between ON and AT. However, as in the case of AN, the obsession with a healthy diet, the ritualized behaviors associated with the preparation and consumption of food, with a selective and narrow interest toward diet, seem to show several overlaps with autistic-like symptomatology. Similarly, the sense of moral superiority and the intolerance toward the eating habits of other people may recall the deficits of the socio-emotional reciprocity typically found in autistic people (20). A previous study by Carpita et al. (15) showed, in a wide University population (students as well as University workers), a greater presence of AT among subjects with ON symptoms, measured by means of the ORTO-15. Moreover, they found that being females and reporting higher AT were statistically predictive factors toward the presence of ON. While this study seems to further support the hypothesis that ON may be associated, like AN, with female presentations of the autism spectrum, the use of ORTO-15, as reported above, was criticized by previous studies.

In this framework, the aim of the present study was to investigate the presence of ON symptoms, measured by means of ORTO-R scores, in a sample of University students with high or low AT, specifically focusing on evaluating the role of gender and of dietary habits in the association between ON and autism spectrum.

## Methods

### Participants

All the students of University of Pisa received an e-mail invitation to participate in the survey. Subjects who agreed to participate were requested to fill out a form for collecting socio-demographic variables (gender, age) and they were subsequently assessed with self-report psychometric instruments. Subjects were also asked to specify the type of diet followed (vegan/vegetarian/omnivorous diet). All the procedures were conducted through an anonymous online form. Students did not receive any payment or benefit for agreeing to participate. The study was conducted in accordance with the Declaration of Helsinki and the local Ethics Committees approved all recruitment and assessment procedures.

### Psychometric Instruments

#### AdAS Spectrum

The Adult Autism Subthreshold Spectrum (AdAS Spectrum) is an instrument tailored to evaluate the presence of full-threshold and subthreshold autism spectrum symptoms and traits, as well as gender-specific traits, in adults with no diagnosis of intellectual disabilities and alterations in language development (41). The questionnaire is composed of 160 items, grouped in 7 domains: *Childhood/adolescence, Verbal communication, Non-verbal communication, Empathy, Inflexibility and adherence to routine, Restrictive interests and rumination, Hyper-hypo reactivity to sensory input*. Higher scores indicate a higher impairment in the assessed dimension. According to the validation study, the AdAS Spectrum was proven to be a reliable quantitative assessment of AT, and it was employed in several clinical and non-clinical studies (21, 33, 34, 42–45). The instrument features also two threshold values: a cut-off score of 70, for identifying the presence of full-blown ASD symptoms, and a cut-off score of 43, for identifying the presence of AT (37).

#### ORTO-R

ORTO-R (19) is the revised and abbreviated version of the ORTO-15 (18), a questionnaire developed to assess orthorexic features. It consists of 6 items: each item can be answered on a Likert scale. Score were reversed with respect to the ORTO-15, according to which lower scores indicated higher ON symptoms. According to ORTO-R scoring system, higher scores suggest an increased tendency toward ON. The ORTO-R was developed through a reassessment of the original data of the ORTO-15 validation study, with the aim to overcome the flaws of the ORTO-15 (19). The latter was one of the first questionnaires in this field and thus was criticized for its lack of reliability and adherence to the actual ON symptoms, while ORTO-R was tailored to be in line with the most recent descriptions and definitions of ON. According to the validation study, the ORTO-R seems to be a promising and reliable tool, showing a McDonald's omega coefficient of 0.75 (19). As in the case of the ORTO-15, the ORTO-R has been developed in Italian and English, while its Italian version has been used for the validation study, performed in an Italian population (18, 19). The questionnaire has also been validated in Arabic (46, 47) and Greek (48), confirming good psychometric properties. A Turkish version has been developed on the basis of the Turkish ORTO-11 (49). In accordance with the procedures followed by previous studies (49), although participants filled out the whole ORTO-15 scale, we proceeded in analyzing only the ORTO-R items, changing and reversing the score in order to report it as ORTO-R score.

The rationale of the use of ORTO-R score lies in the fact that this instrument has been developed with the aim to overcome the issues reported in the scientific literature about the ORTO-15 (19). While other instruments have been developed for measuring ON (50), the use of the ORTO-R may allow evaluating ON symptoms with a new and improved version of one of the first and more used instruments in the field, and, as a consequence, may be considered as a point of strength and novelty with respect to studies which used the old version of the scale.

### Statistical Analyses

For the aims of the present work we compared two groups of students, splitting the sample on the basis of the presence of significant AT according to the AdAS Spectrum (high AT and low AT group). In particular, students with a AdAS Spectrum score beyond the cut-off of 43 for significant AT were included in the high AT group, while subjects who scored below were included in the low AT group. Student's *t*-tests and Chi-square tests were employed to compare sociodemographic variables and ORTO-R scores between groups. In order to confirm the reliability of the ORTO-R as used in this study, we calculated Cronbach's alpha coefficient for estimating its internal consistency. In our sample, the ORTO-R showed a good internal consistency, with a Cronbach's alpha of 0.740. Moreover, Student's *t*-tests were used to compare ORTO-R scores among subjects with different sociodemographic characteristics. Pearson's correlation coefficient was calculated for investigating the presence of significant correlations between the AdAS Spectrum and the ORTO-R. We performed a decision tree model in order to identify which variables among gender, type of diet, and presence of AT best predicted ORTO-R scores. This analysis allows us to examine interactions among variables and create a decision tree model, graphically representing the findings as an inverted tree. The model starts with a root node with all the cases included, and then the tree grows by choosing at each step the independent variable with the higher interaction with the dependent variable. The model also merges the categories defined by predictors when no difference is reported with respect to the dependent variable. Finally, an analysis of variance (ANOVA) with a factorial design was performed with ORTO-R as dependent variable and the presence of high AT and gender as independent variables. All the analyses were conducted with SPSS, version 24 (51).

## Results

On the basis of the AdAS Spectrum cut-off, we identified two groups: subjects with high AT (high AT group: 61.59%; *N* = 1,318) and a group subjects with low AT (low AT group: 38.41%; *N* = 822). The mean age of the sample was 23.80 ± 4.80. The sample was composed by a total of 2,140 students, 66.1% (*N* = 1,414) identified themselves as females and 33.9% (*N* = 726) as males. Moreover, 1,956 students (94.3%) followed an omnivorous diet, while 118 students (5.7%) were following at the time of the questionnaire a vegetarian or vegan diet. No significant differences were found with respect of gender and type of diet distribution between high AT and low AT group. When performing Student's *t*-tests, we found that subjects in the high AT group showed a significantly higher ORTO-R score than the low AT group (see Table 1). Moreover, in the overall sample females reported significantly higher ORTO-R scores than males (13.31 ± 3.63 vs. 11.57 ± 3.24; *t* = −11.32; *p* < 0.001), while subjects who followed a vegetarian/vegan diet showed significantly higher ORTO-R score than those following an omnivorous diet (14.19 ± 3.40 vs. 12.63 ± 3.59; *t* = −4.58; *p* < 0.001).

**Table 1:** Comparison of sociodemographic variables and ORTO-R scores between high AT and low AT groups.

		***High AT group (N = 1,318)***	***Low AT group (N = 822)***	***t***	***p***
		***mean ±SD***	***mean ±SD***	
**ORTO-R**		13.30 ± 3.67	11.79 ± 3.26	−9.95	**<0.001**
		*N (%)*	*N (%)*	***Chi-square***	***p***
**Gender**	F	855 (64.9)	559 (68.0)	2.22	0.136
	M	463 (35.1)	263 (32.0)		
**Diet^°^**	Omnivorous	1,195 (93.7)	761 (95.2)	2.11	0.146
	Vegetarian/ Vegan	80 (6.3)	38 (4.8)		

° *Only a subset of 2,074 students reported this data. Significant p values are reported in bold*.

When performing a Pearson's correlation coefficient, we also found that ORTO-R was significantly correlated with the AdAS Spectrum in both females and males, although correlation coefficients were slightly higher among females for total AdAS Spectrum and most of the domain scores (see Table 2).

**Table 2:** Correlations between ORTO-R and AdAS Spectrum scores among males and females.

	**ORTO-R**	
	**Males (*N* = 726)**	**Females (*N* = 1,414)**
**AdAS spectrum**
Childhood/Adolescence	0.165^*^	0.210^*^
Verbal communication	0.187^*^	0.147^*^
Non-verbal communication	0.229^*^	0.272^*^
Empathy	0.090^*^	0.125^*^
Inflexibility and adherence to routine	0.227^*^	0.266^*^
Restricted interests and rumination	0.230^*^	0.244^*^
Hyper-Hypo reactivity to sensory input	0.198^*^	0.175^*^
AdAS Spectrum total score	0.243^*^	0.274^*^

* *p <0.001*.

**Figure 1:**
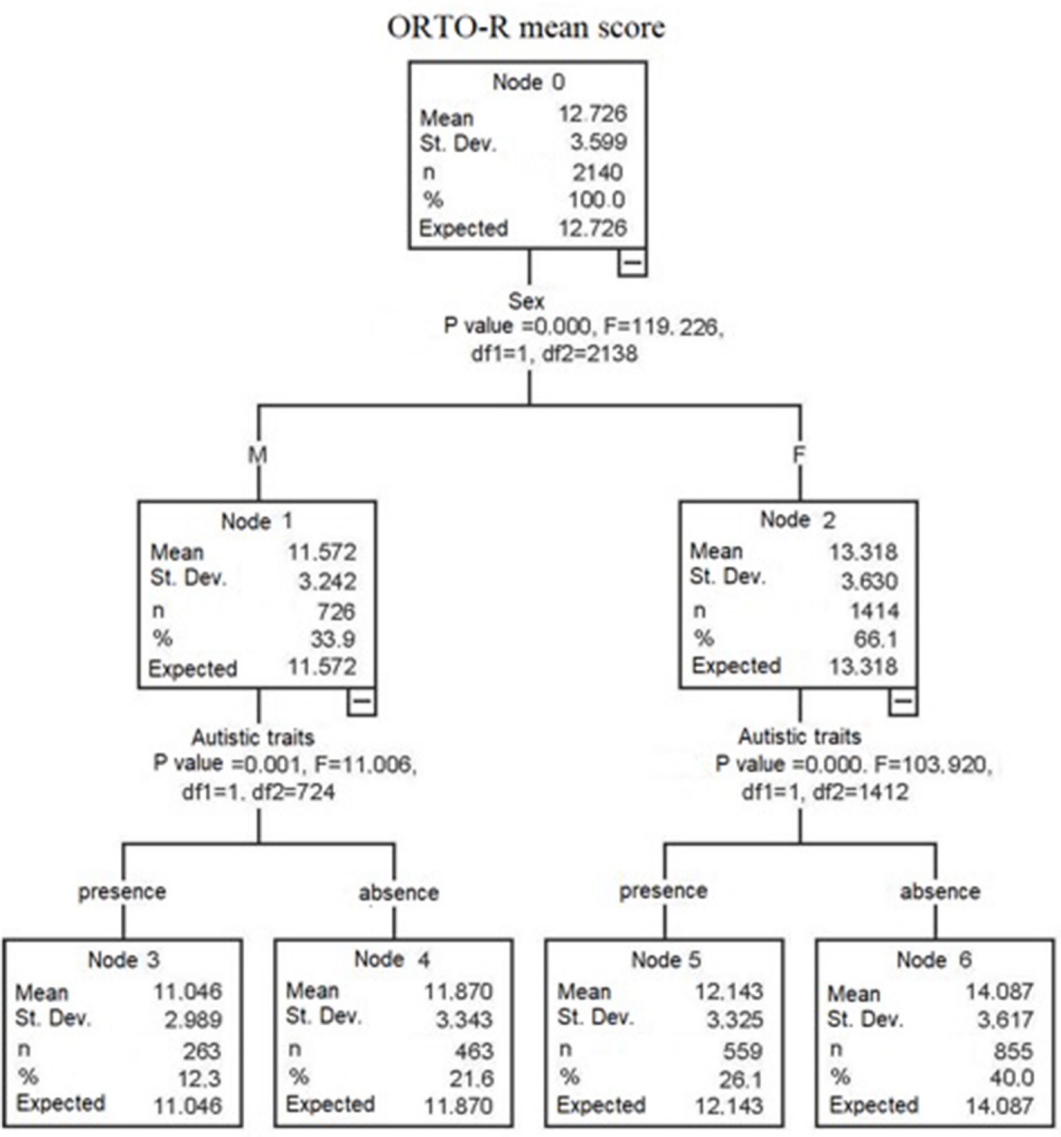
Tree decision model with ORTO-R score as independent variable and gender, type of diet and presence of high/low AT as independent variables.

**Figure 2:**
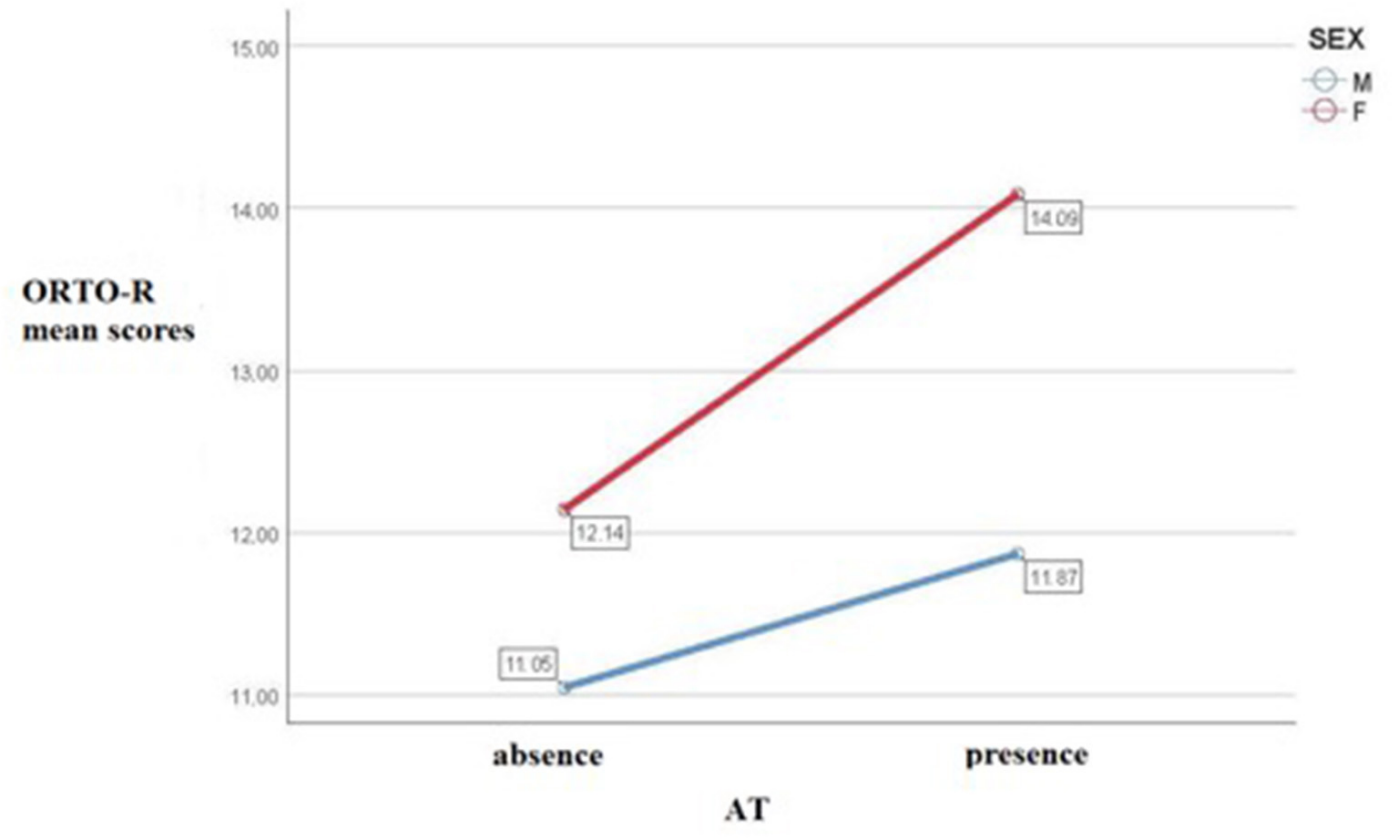
Profile plot of estimated marginal means for ORTO-R depending from presence of high/low AT in males and females.

Results from the decision tree model, with gender, type of diet and presence of high AT as independent variables and ORTO-R score as dependent variable, showed in the first step the presence of significantly higher ORTO-R scores among females than among males. Subsequently, among both females and males, the high AT group scored higher than the no AT group. No effect was found for the type of diet (see Figure 1). Finally, in order to further deepen the investigation on the effect and the interaction between the presence of AT and gender on ORTO-R scores, we performed a factorial ANOVA analysis. Results reported a significant main effect of gender and of AT on ORTO-R score: [*F*_(1, 2136)_ = 105.79, *p* < 0.001] and [*F*_(1, 2136)_ = 73.83, *p* < 0.001], respectively. There was also a significant interaction between gender and AT on ORTO-R score: [*F*_(1, 2136)_ = 12.06, *p* = 0.001]. In particular, we found that, although in both genders ORTO-R scores increased with the increase of the AdAS Spectrum scores, the increasing trend was higher among females (see Table 3 and Figure 2).

**Table 3:** Factorial ANOVA analysis with gender and presence of high/low AT as independent variables and ORTO-R score as dependent variable.

**Source**	**Type III sum of squares**	***df***	**Mean square**	***F*_(1, 236)_**	***p***
Corrected model	2854.16	3	951.39	81.77	**<0.001**
Intercept	270755.07	1	270755.07	23271.24	**<0.001**
Gender	1230.87	1	1230.87	105.79	**<0.001**
AT	859.03	1	859.03	73.83	**<0.001**
Gender *AT	140.28	1	140.28	12.06	**0.001**
Error	24851.82	2136	11.64	-	**-**
Total	374265.00	2140	-	-	**-**
Corrected total	27705.99	2139	-	-	**-**

*R squared = 0.103; Adjusted R squared = 0.102. Significant p values are reported in bold*.

## Discussion

The aim of the present study was to assess the presence of ON in a population of University students, with high and low AT, with a focus on the role of diet and gender differences. Firstly, it should be noted that in our sample a high number of subjects (61.59%; *N* = 1,318) reported significant levels of subthreshold AT according to the AdAS Spectrum, being thus included in the “high AT group.” This data may be in line with the previous reports of an increased presence of high AT among University students, especially those enrolled in scientific courses and/or in highly selective Universities (29, 43). However, due to the kind of recruitment procedures, it might also be hypothesized a selection bias in the sample, with a higher participation of subjects more interested in the topic. Our data support the reliability of the ORTO-R as a measure for ON symptoms, reporting for the instrument a good internal consistency as measured by Cronbach's alpha coefficient. The value of 0.740 reported in our sample is in line with those reported for the ORTO-R in other languages (46–48) and with the measures of reliability reported in the original validation study (19).

We found a significantly higher prevalence of ON symptoms among subjects with high AT, among women and among participants following a vegetarian/vegan diet. Despite some authors did not found significant gender differences in ON, or even reported a higher prevalence among men (10, 11, 18, 52, 53), our results seem in line with the studies which instead reported a higher prevalence of ON among females (14, 54, 55). Dell'Osso et al. previously highlighted a higher prevalence of ON among females and among subjects with a vegetarian/vegan diet also in another sample of University students (5, 6). As previously stressed, the increased prevalence of ON among females supported the hypothesis of a possible continuum between ON and AN, which might also share a similar distribution among genders (6, 14, 15). Regarding the role of diet, our results confirm previous reports from the literature, which highlighted a link between ON, obsession for healthy eating and vegan/vegetarian dietary habits (6, 14, 15, 40, 56, 57). In particular, in a recent review from Brytek-Matera et al. (58), an association between vegetarianism and eating behaviors typical of ON was found in 11 out of the 14 studies included, thus raising the question if vegetarianism may represent a risk factor for the development of ON.

The higher prevalence of ON symptoms in subjects with high AT reported in this study is in line with the higher levels of AT among subjects scoring over the threshold for ON (as measured by the ORTO-15) previously reported in a University population (15). The similar findings reported by means of different questionnaires strengthen the validity of the association between these two conditions. Our results further support the link between ON and the autism spectrum, according to the spreading hypothesis of an association between ASD and restrictive eating disorders (20–22, 59–62). Noticeably, subjects with ASD often display restrictive eating behaviors, such as highly selective food choices or a total refusal of specific kinds of food (20, 63), a feature that may resemble the habits of orthorexic subjects. At the same time, subjects affected by ON typically show ritualized behaviors in the preparation and consumptions of food and often report a strong cognitive focus on eating habits, which recall the well-known stereotyped behaviors and interests of ASD. In addition, the feeling of moral superiority and the intolerance toward eating behaviors of other people may suggest the presence of autistic-like deficits in socio-emotional reciprocity in ON subjects (2, 20).

A significant correlation was also found between ORTO-R and the AdAS Spectrum, with a tendency toward stronger correlation coefficients among females. Moreover, according to our results, there was a significant effect of the interaction between AT and gender on ON: while ORTO-R scores rose with the increase of AdAS Spectrum scores in both genders, the rising of the ORTO-R scores was higher among females. These findings, suggesting a specifically higher association between ON and autism spectrum among females, may be considered in line with the literature which hypothesized a link between AN and female presentations of ASD, leading to hypothesize that the overlap between autism and eating disorder spectra among females may extend also to ON (14, 64). According to this hypothesis, ON may be included in the same autism spectrum phenotype of AN and, subsequently, AN and ON might share the same autistic core which, in this case, would be expressed by an inflexible and narrow focus on eating habits, together with stereotyped and repetitive behaviors related to food and altered socio-emotional reciprocity.

Several hypotheses were reported for explaining the link between autism spectrum and restrictive eating disorders. As reported above, increasing literature is suggesting a possible reconceptualization of AN as a gender-specific presentation of ASD (14). According to this hypothesis, ASD and AN (and, eventually, also ON) may share a similar pathophysiology. Starting from the same kind of neurodevelopmental impairment, the psychopathological trajectory would lead to partially different clinical pictures due to the interaction with other biological and genetic factors (particularly sex) and, eventually, with gender-related socio-cultural factors (14, 62). Conversely, other authors hypothesized that autistic features in patients FED may be a consequence of the chronic starvation (20, 21). Another issue that should be clarified is the later onset of AN/ON, while selective eating food in ASD is typically present since childhood. However, it should be noted that FED external manifestations were known to be influenced by the socio-cultural context: in this framework, and considering the higher tendency toward camouflaging behaviors often reported among females in the autism spectrum, it might be possible that the specific mentalization and verbalization of the altered eating behaviors would vary from childhood to later stages of life, when the subjects may be more exposed to (and influenced by) socio-cultural and environmental factors (14, 20, 21).

In conclusion, the specific nature of the association between ASD and restrictive eating disorders, including ON, need to be further clarified. The hypothesis of a shared neurodevelopmental alteration, with gender-specific differences in clinical presentation, is gaining attention in the literature, but further evidence from different fields (neurobiology, genetics, psychopathology) are needed to confirm or discard it.

This study should be considered in light of several limitations. Firstly, our sample included only University students, and thus our results could not be extended to the general population. Secondly, subjects were recruited on a voluntary basis, eventually leading to selection biases in the sample (e.g., recruitment of subjects more interested in autism related problems/eating habits). Moreover, both the AdAS Spectrum and ORTO-R are self-reported questionnaires, and the use of this kind of instruments may lead to biases in the results due to an underestimation or an overestimation of the symptoms by the subjects. In order to limit dropouts during the filling out of the questionnaires, we also chose to use only one instrument for each condition, but this choice may have limited the accuracy of the assessment, in particular in the case of ON: the ORTO-R is a short instrument, and this feature may have prevented us from reaching a better and comprehensive assessment of a complex condition as ON. In addition, no information was available about the presence among the subjects of an actual diagnosis of ASD or of Feeding and eating disorders, or also of other comorbid mental disorders, which may have affected our results. Finally, this study featured a cross-sectional design, and subsequently it was not possible to make inferences on any cause-effect or temporal relationship regarding our findings. Further studies featuring a full-fledged clinical assessment and possibly, a longitudinal design are warranted for clarifying the relationship between ON and AT.

## Conclusion

Globally, results from this work seems to confirm the higher prevalence of ON symptoms among females, and the link between ON and a specific interest toward dietary choices. In addition, in line with some previous studies, our findings highlighted an association between AT and ON, stressing that this association may be stronger among females. In the broader framework of the overlap between AN and autism spectrum conditions reported in the recent literature, our study might support the hypothesis of a possible reconceptualization of restrictive eating disorders as gender-specific presentations of the autism spectrum, in light of a possible continuum and shared pathophysiology between AN and ON.

## Data Availability Statement

The raw data supporting the conclusions of this article will be made available by the authors, without undue reservation.

## Ethics Statement

The studies involving human participants were reviewed and approved by Comitato Bioetico dell'Università di Pisa, University of Pisa, Pisa, Italy. The patients/participants provided their written informed consent to participate in this study.

## Author Contributions

All authors gave substantial contribution to the study and approved the final version of the manuscript.

## Conflict of Interest

The authors declare that the research was conducted in the absence of any commercial or financial relationships that could be construed as a potential conflict of interest.

## Publisher's Note

All claims expressed in this article are solely those of the authors and do not necessarily represent those of their affiliated organizations, or those of the publisher, the editors and the reviewers. Any product that may be evaluated in this article, or claim that may be made by its manufacturer, is not guaranteed or endorsed by the publisher.
